# Measurement of total liver blood flow in intact anesthetized rats using ultrasound imaging

**DOI:** 10.1002/prp2.731

**Published:** 2021-03-04

**Authors:** Christopher R. Gibson, Alexa Gleason, Eric Messina

**Affiliations:** ^1^ Departments of Pharmacokinetics Pharmacodynamics and Drug Metabolism (CRG) Translational Biomarkers (AG, EM) Merck & Co., Inc. West Point PA USA

**Keywords:** blood flow, liver, rat, ultrasound

## Abstract

This short report describes the measurement of total liver blood flow in commonly used laboratory rats using the relatively non‐invasive approach of ultrasound imaging. A total of 29 rats (*n* = 26 Wistar‐Han, *n* = 3 Sprague–Dawley) were imaged and both male and female rats were included. The mean (SD) total liver blood flow of all animals combined was 33.3 ± 7.8 mL/min, or 104.3 ± 17.1 mL/min/kg when normalized to observed body weight at the time of imaging. There was a trend for higher unnormalized total liver blood flow as body weight increased and the female rats had, in general, the lowest body weight and total liver blood flow of the animals studied. There were no major differences in total liver blood flow between the small number of Sprague–Dawley rats used in the study and the larger Wistar‐Han group. Further research would be needed to accurately characterize any subtle differences in body weight between rats of different strains, sexes, and body weight.

## Graphical abstract

Mean (SD) total liver blood flow as measured by ultrasound imaging in female and male rats separately and combined (Panel A). The mean total liver blood flow for both sexes combined was 33.3 (7.8) mL/min. Mean (SD) body weight‐normalized total liver blood flow as measured by ultrasound imaging in female and male rats separately and combined (Panel B). The mean body weightnormalized total liver blood flow for both sexes combined was 104.3 (17.1) mL/min/kg.

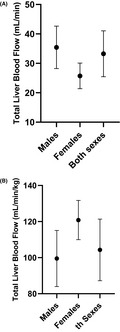

**Abbreviations**
BW: body weight
ECG: electrocardiogram
IP: intraperitoneal
PBPK: physiologically based pharmacokinetic models
PKPD: pharmacokinetic‐pharmacodynamic models

**Significance Statement**

This short communication describes the measurement of total liver blood flow in commonly used laboratory rat strains using a relatively non‐invasive imaging technique. The data collected suggest body weight‐normalized liver blood flow is higher than previously measured ex vivo using invasive surgical techniques and may be useful to help scientists interpreting PK data from IV studies in rats or as a parameter in physiologically based PKPD models.

## 1 INTRODUCTION

The liver is a vital organ that is largely responsible for the elimination of a wide range of xenobiotic therapeutics including small molecules, peptides, and some biologics. Translational approaches have been developed throughout the years to integrate experimental data on drug metabolism, distribution, and transport with the relevant in vivo physiology to model and predict basic pharmacokinetic concepts like clearance which can influence dose and dose regimen. Clearance is the irreversible removal of drug molecules from the site of measurement, which typically is from the systemic circulation.^1^ Considering the predominant role the liver plays as a site of drug elimination from the body, having reliable approaches to model and predict hepatic drug clearance becomes an important activity for new drug discovery research. Approaches such as the well‐stirred model of hepatic extraction relate drug‐specific parameters like intrinsic clearance, blood‐to‐plasma partitioning, and plasma protein binding to physiological parameters which are species‐specific and generally independent of the drug such as liver blood flow.^1^, ^2^, ^3^ Accordingly, having reliable and trustworthy quantitative estimates of the physiological parameters, like liver blood flow, can help enable accurate modeling and prediction of hepatic drug clearance.

Rats are a commonly used nonclinical animal model in biomedical research, including in the discovery and development of new medicines. Studies to date measuring liver blood flow in laboratory rats have all used techniques requiring animals to be anesthetized and surgically prepared with multiple indwelling cannula and sensors being strategically placed into the animal allowing for the injection of radiolabeled microspheres which become occluded in the capillary bed of the liver.^4^, ^5^, ^6^, ^7^ Careful simultaneous removal of blood samples was required during the microsphere injection all while attempting to keep overall cardiac output and blood pressure unchanged. The animals were subsequently sacrificed following the procedure and liver blood flow was estimated ex vivo using mass balance principles, assuming the microspheres were well‐mixed upon injection, by measuring the radioactivity in the liver. Additionally, many of the studies were investigating how different experimental treatments, such as daily intraperitoneal (IP) injection of enzyme inducers or placebo, would affect liver blood flow meaning the animals were subject to additional experimental manipulations beyond the invasive surgery needed for the microsphere technique. With these caveats and assumptions, the liver blood flow in rats was estimated to be approximately 52–67 mL/min/kg.^5^, ^6^, ^7^

Given the importance of knowing the quantitative value of total liver blood flow in laboratory rats, we took the opportunity to experimentally measure it using the non‐invasive technique of ultrasound imaging. Ultrasound uses high‐frequency sound waves, generated and received by a transducer, to measure various biomarkers like blood vessel diameter and blood velocity in vivo. These clinically relevant biomarkers are often used to monitor hemodynamic changes in blood vessels. In general, images are generated by the differences in reflected sound waves as they pass through various tissues back to the transducer. Blood velocity can be measured based on the Doppler shift of blood as it passes through the vessel lumen using pulsed wave Doppler mode. Preclinical ultrasound equipment is ideally suited for small animal studies because it has an axial resolution of approximately 30 µm, allowing for precise and reproducible measurement of vessels as small as 200 µm in rodent experiments. The main advantage to using ultrasound for this analysis was that the data were acquired fairly quickly without any significant chemical or surgical intervention. Ultrasound imaging has a well‐documented history being used to acquire hemodynamic changes in blood vessels in preclinical^8^, ^9^ and clinical studies.^10^, ^11^ Data from these studies can be used to reliably calculate blood flow by measuring changes in lumen diameter and blood velocity of the main vessels that supply blood to the liver.^12^

## 2 METHODS

### 2.1 Ethics statement

The measurements described below have been conducted in accordance with the Guide for the Care and Use of Laboratory Animals as adopted by the U.S. National Institutes of Health, and were approved by the Institutional Animal Care and Use Committee (IACUC). The measurements were conducted in accordance with all the animal care and use laws, regulations, and guidelines.

### 2.2 Animals

A total of 29 rats from 12–40 weeks of age were used for ultrasound imaging (Taconic Biosciences Inc). There were 26 Wistar Han (22 male/4 female) and 3 Sprague Dawley (2 males/1 female) rats used for the measurements. Animals were housed in microisolator cages, provided standard chow/water and maintained on 12‐h light/dark cycles in an HEPA‐filtered environment. The liver blood flow measurements were obtained in an opportunistic manner as the rats were being used for other research purposes. However, none of the animals used received any experimental or other treatments prior to liver blood flow measurement. Four animals were imaged at three different time points, and 12 animals were imaged at two different time points to capture changes in blood flow due to changes in body weight. Intervals between image timepoints were different for each animal due to the opportunistic manner of these measurements.

### 2.3 Measurement of liver blood flow using ultrasound imaging

A Vevo 2100 or Vevo 3100 Ultrasound System (FUJIFILM VisualSonics, Inc.), with a linear array transducer (MS400) and a center frequency of 30 MHz was used for all image acquisition. Animals were weighed immediately prior to every imaging timepoint. Animals were anesthetized using isoflurane (Zoetis Inc. Kalamazoo, MI) at approximately 1.5% concentration supplied by medical air through a vaporizer to maintain animals at 60 ± 5 breaths per minute for the duration of the measurement (approximately 10 min). They were positioned supine on a heated platform (VSI) equipped with an integrated temperature sensor and ECG electrodes for monitoring heart and respiratory rate.

Fur was removed over the region of interest using a #50 clipper blade. Prior to imaging, a warm acoustic gel (Aquasonic 100, Parker Laboratories) was applied to the skin to facilitate ultrasound transmission. Portal vein and hepatic artery imaging was performed at the region of the hilum of the liver. The transducer was positioned in a longitudinal orientation to the vessels of interest to facilitate the acquisition of various Bmode, Mmode, and PW Doppler images.

### 2.4 Analysis/statistics

Ultrasound image analysis was performed using VisualSonics Vevo Lab v1.7.0 and v3.2.0 software. Measurements taken included portal vein diameter and velocity, and hepatic artery diameter and velocity. Individual vessel flow was captured using the following equation: Flow = (cross‐sectional area x mean velocity)/BW. Total liver blood flow was calculated using the sum of the blood flows from the hepatic artery and portal vein. Statistical and graphical analysis of the data was performed using Microsoft Excel and GraphPad Prism (Version 8.1.1 for Windows, GraphPad Software, www.graphpad.com). Values are reported as arithmetic mean ± standard deviation.

## 3 RESULTS

**FIGURE 1:**
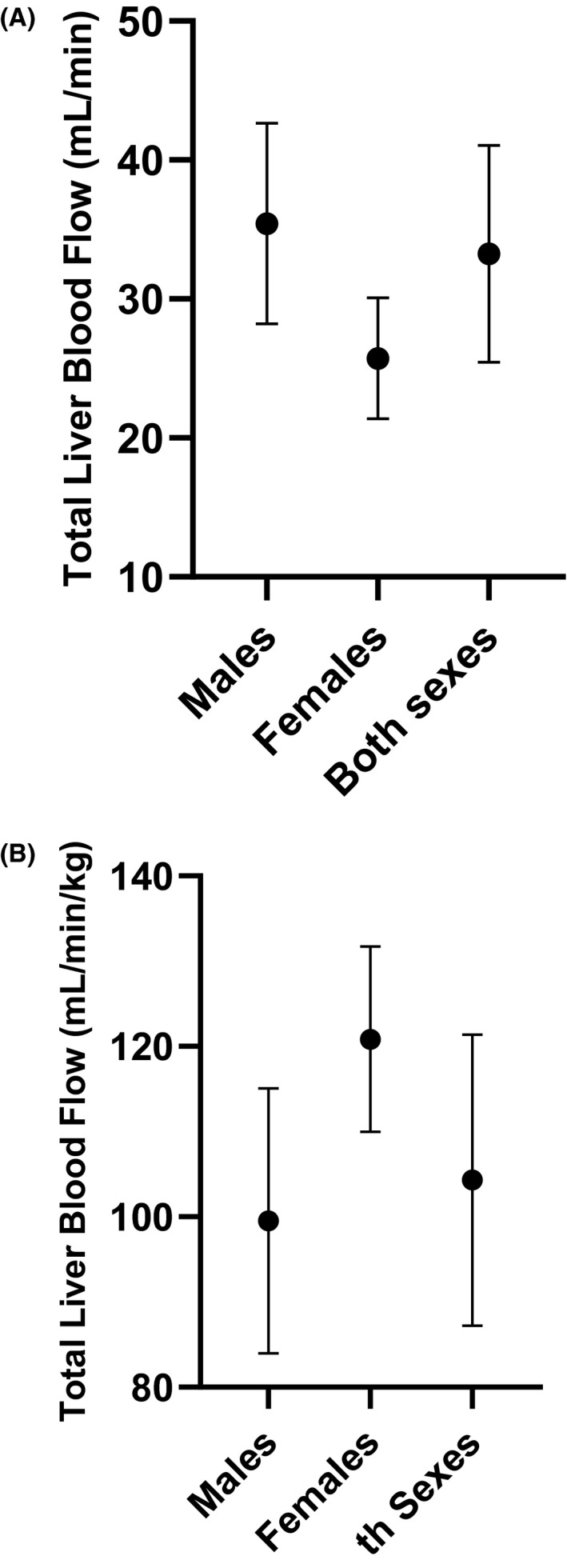
(A) Mean (SD) total liver blood flow as measured by ultrasound imaging in female and male rats separately and combined. The mean total liver blood flow in female rats was 25.7 (4.4) mL/min and the mean in male rats was 35.4 (7.2) mL/min. The mean total liver blood flow for both sexes combined was 33.3 (7.8) mL/min. (B) Mean (SD) body weight‐normalized total liver blood flow as measured by ultrasound imaging in female and male rats separately and combined. The mean body weight‐normalized total liver blood flow in female rats was 120.8 (10.9) mL/min/kg and the mean in male rats was 99.5 (15.6) mL/min/kg. The mean body weight‐normalized total liver blood flow for both sexes combined was 104.3 (17.1) mL/min/kg

**FIGURE 2:**
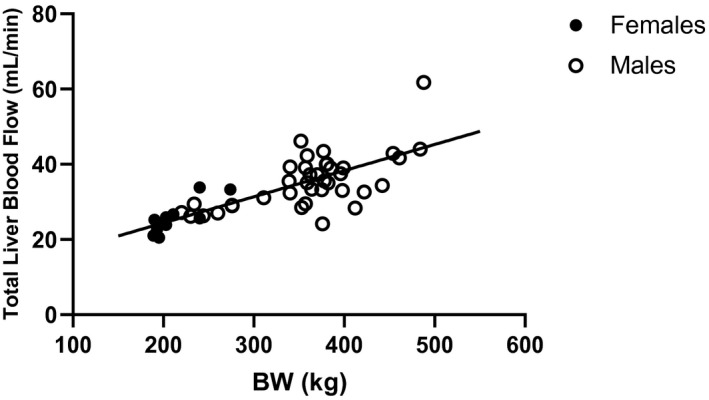
Individual observed total liver blood flow in male (open circles) and female (filled circles) rats versus observed body weight at the time of ultrasound imaging. Linear regression line shown (*p* < 0.0001)

The mean (SD) total liver blood flow in male and female rats individually and combined is shown in Figure 1 and Table 1. Body weight‐normalized liver blood flows were also calculated for comparison. The mean total liver blood flow in male rats was 35.4 ± 7.2 mL/min, whereas the average in female rats was slightly lower at 25.7 ± 4.4 mL/min. The mean total liver blood flow of both male and female groups combined was 33.3 ± 7.8 mL/min. The mean body weight‐normalized total liver blood flow in male rats was 99.5 ± 15.6 mL/min/kg and was 120.8 ± 10.9 mL/min/kg in female rats. The mean body weight‐normalized total liver blood flow in both male and female rats combined was 104.3 ± 17.1 mL/min/kg. Of note, the body weight‐normalized liver blood flow in female rats was higher than that observed in males, opposite to what was observed when considering the unnormalized measured flow rates. The individual measured total liver blood flows, for each sex, are plotted versus body weight at the time of measurement and shown in Figure 2. There was a statistically significant linear correlation between liver blood flow and body weight (*Y* = 0.06951 * BW + 10.51, *p* < 0.0001). Female rats had, in general, lower body weight relative to males used in the study.

**TABLE 1:** Individual data for total liver blood flow measurements in intact anesthetized rats using ultrasound imaging

Animal ID	Sex	Strain	Body weight at time of measurement (g)	Total liver blood flow (mL/min)	Total liver blood flow (mL/min/kg)
F898	F	WH	193	22.7	117.5
F898	F	WH	203	23.9	117.8
F897	F	WH	190	25.2	132.8
F897	F	WH	189	21.0	111.2
F896	F	WH	195	20.5	105.2
F896	F	WH	200	24.2	121.1
F896	F	WH	240	33.8	141.0
F697	F	SD	274	33.3	121.6
F895	F	WH	203	25.9	127.6
F895	F	WH	211	26.7	126.6
F895	F	WH	240	25.6	106.8
M894	M	WH	234	29.4	125.7
M894	M	WH	276	29.0	105.0
M894	M	WH	340	39.3	115.6
M893	M	WH	220	27.1	123.4
M893	M	WH	260	27.0	103.9
M893	M	WH	340	32.4	95.2
M699	M	WH	230	26.1	113.7
M699	M	WH	276	29.1	105.5
M700	M	WH	244	26.3	107.7
M700	M	WH	311	31.1	100.0
M693	M	WH	488	61.7	126.5
M694	M	WH	484	44.0	90.9
M685	M	SD	454	42.9	94.5
M686	M	SD	461	41.7	90.4
82	M	WH	353	28.5	80.8
82	M	WH	382	35.1	91.8
86	M	WH	362	37.2	102.8
86	M	WH	412	28.4	68.8
88	M	WH	399	39.1	97.9
88	M	WH	442	34.4	77.7
90	M	WH	370	37.3	100.7
90	M	WH	396	37.5	94.7
92	M	WH	339	35.5	104.6
92	M	WH	398	33.0	82.9
96	M	WH	357	29.5	82.6
96	M	WH	376	24.2	64.3
98	M	WH	357	39.1	109.6
98	M	WH	381	40.1	105.2
112	M	WH	380	40.0	105.2
112	M	WH	422	32.6	77.3
84	M	WH	359	35.1	97.8
100	M	WH	364	33.3	91.6
102	M	WH	378	35.6	94.1
104	M	WH	352	46.1	131.1
106	M	WH	385	38.9	101.2
108	M	WH	375	33.2	88.6
110	M	WH	377	43.4	115.1
114	M	WH	359	42.3	117.8

Abbreviations: F, females; M, males; SD, Sprague–Dawley; WH, Wistar‐Han.

## 4 DISCUSSION

The trends in both body weight‐normalized and unnormalized total liver blood flow between rat sexes and different body weights were noteworthy. In general, female rats had lower measured total liver blood flow, but higher body weight‐normalized total liver blood flow compared to males (Figure 1). It is apparent when looking at the individual data in Figure 2 that the female rats included in the study were at the lower end of the body weight range of the rats studied. This could explain the observed trend in normalized and non‐normalized liver blood flow between sexes since the difference in body weight was greater than the difference in underlying measured liver blood flow between the sexes. As for differences between strains, of the 29 rats studied 3 were Sprague‐Dawley (2 M/1F) and the remainder were all Wistar‐Han. The measured total liver blood flow in Sprague–Dawley rats was within ±2 × SD of the mean values estimated from all the animals, suggestive of no major differences in liver blood flow between strains. More research would be needed to accurately characterize any subtle differences in liver blood flow between rats of different strains, sexes, and body weights.

The values reported here are higher than those which were previously measured ex vivo using the invasive surgical radioactive microsphere techniques which ranged 52–67 mL/min/kg.^5^, ^6^, ^7^ And although the rats included in this study required anesthesia, we believe the measurements reported here more closely reflect the actual liver blood flow in an intact conscious rat relative to values obtained ex vivo following significant experimental and surgical manipulation. Experience using isoflurane as an anesthetic in rodents at Merck Research Laboratories suggests it allows for the maintenance of a stable respiration and heart rate in rats over the relatively short time frame needed to perform the ultrasound imaging. Similarly, isoflurane administration to dogs did not change the liver blood flow.^13^ So while we cannot be certain that the values we have observed are a better estimate of in vivo liver blood flow in a conscious unrestrained rat relative to values already reported, it is reasonable to assume that a technique requiring significantly less manipulation of the animal (e.g., not undergoing surgical procedures) would produce a more accurate value.

One potential use of these newly measured values would be contextualizing the role of potential extra‐hepatic clearance mechanisms when interpreting total body clearance obtained from intravenous pharmacokinetic studies of small molecules in rats. Clearance is an important compound‐specific pharmacokinetic parameter that is determined, in part, by total liver blood flow.^1^, ^2^, ^3^ It can be important to contextualize observed blood or plasma total body clearances obtained from in vivo pharmacokinetic studies to the overall liver blood flow of the species studied. For example, according to the commonly accepted well‐stirred model of hepatic extraction, in vivo total body blood clearances in excess of liver blood flow may indicate that additional organs beyond the liver are involved in the elimination of the compound. Such context can be used during candidate drug optimization efforts to help understand and manipulate specific mechanisms of drug clearance to help design the best compounds in terms of dose and dose regimen for the intended patient population.

Additionally, the liver blood flows we have measured, when integrated with other in vitro and in silico data/parameters, can be used for the building and usage of translational approaches involving physiologically based pharmacokinetic modeling (PBPK). PBPK models are mathematical representations of physiology, anatomy, and biochemistry and have a number of uses in the pharmaceutical and toxicological sciences.^14^ Physiological parameters like organ weight, tissue composition, and organ blood flow are used in PBPK models for activities such as prediction of pharmacokinetics and pharmacodynamics (PKPD), drug‐drug interactions, tissue exposure, and dose.^15^, ^16^ As an example in computational toxicology, Sharma and colleagues used a PBPK model to translate flutamide pharmacokinetics, metabolism, and tissue exposures in rats to those observed and anticipated in humans to assess the risk of flutamide as an environmental pollutant.^17^ In their work, the rat liver blood flow was estimated indirectly using reported cardiac output in rat, and the estimated percentage of cardiac output flowing to the liver and was approximately three‐fold greater than the value we measured.^17^ In spite of this, the work provides a nice example of how in vitro and non‐clinical data can be incorporated into a translational model to provide human context. Having accurate quantitative knowledge of the underlying physiological parameters involved in drug distribution and elimination, such as liver blood flow, enables accurate modeling and improves the ability to make the best decisions when considering the model output. And direct measurement of parameters like liver blood flow using non‐invasive approaches, like what we report here, will help support the construction of quality physiological models resulting in the best decisions possible and reduce some of the ambiguity and variability in the value of rat liver blood flows used for physiologically based modeling.

## CONFLICT OF INTEREST

Christopher Gibson, Eric Messina, and Alexa Gleason are all employees of Merck and Co., Inc.

## AUTHOR CONTRIBUTIONS

Participated in research design: Gibson, Messina, and Gleason. Conducted experiments: Messina and Gleason. Performed data analysis: Gibson, Messina, and Gleason. Contributed to writing manuscript: Gibson, Messina, and Gleason.
